# A Qualitative Assessment of Provider and Client Experiences With 3- and 6-Month Dispensing Intervals of Antiretroviral Therapy in Malawi

**DOI:** 10.9745/GHSP-D-19-00286

**Published:** 2020-03-30

**Authors:** Julie Hubbard, Khumbo Phiri, Corrina Moucheraud, Kaitlyn McBride, Ashley Bardon, Kelvin Balakasi, Eric Lungu, Kathryn Dovel, Gift Kakwesa, Risa M. Hoffman

**Affiliations:** a University of California Los Angeles, David Geffen School of Medicine, Division of Infectious Diseases, Los Angeles, CA, USA.; b Partners in Hope Medical Center, Lilongwe, Malawi.; c University of California Los Angeles, Fielding School of Public Health, Department of Health Policy and Management, Los Angeles, CA, USA.; d University of Washington, School of Public Health, Department of Epidemiology, Seattle, WA, USA.

## Abstract

**Introduction:**

Multimonth dispensing (MMD) of antiretroviral therapy (ART) is a differentiated model of care that can help overcome health system challenges and reduce the burden of HIV care on clients. Although 3-month dispensing has been the standard of care, interest has increased in extending refill intervals to 6 months. We explored client and provider experiences with MMD in Malawi as part of a cluster randomized trial evaluating 3- versus 6-month ART dispensing.

**Methods:**

Semi-structured in-depth interviews were conducted with 17 ART providers and 62 stable, adult clients with HIV on ART. Clients and providers were evenly divided by arm and were eligible for an interview if they had been participating in the study for 1 year (clients) or 6 months (providers). Questions focused on perceived challenges and benefits of the 3- or 6-month amount of ART dispensing. Interviews were transcribed, and data were coded and analyzed using constant comparison.

**Results:**

Both clients and providers reported that the larger medication supply had benefits. Clients reported decreased costs due to less frequent travel to the clinic and increased time for income-generating activities. Clients in the 6-month dispensing arm reported a greater sense of personal freedom and normalcy. Providers felt that the 6-month dispensing interval reduced their workload. They also expressed concerned about clients' challenges with ART storage at home, but clients reported no storage problems. Although providers mentioned the potential risk of clients sharing the larger medication supply with family or friends, clients emphasized the value of ART and reported only rare, short-term sharing, mostly with their spouses. Providers mentioned clients' lack of motivation to seek care for illnesses that might occur between refill appointments.

**Conclusions:**

The 6-month ART dispensing arm was particularly beneficial to clients for decreased costs, increased time for income generation, and a greater sense of normalcy. Providers' concerns about storage, sharing, and return visits to the facility did not emerge in client interviews. Further data are needed on the feasibility of implementing a large-scale program with 6-month dispensing.

## Abstract (precis)

Clients with HIV on antiretroviral therapy (ART) perceived the 6-month ART dispensing interval as highly acceptable due to reduced transport costs and increased time for income-generating activities. Providers reported benefits in reduced clinic workload and improved ability to see clients who need more support. Before implementing this dispensing interval on a large scale, countries should conduct further research on how to encourage client health-seeking behaviors for health problems, ensure women have access to family planning services outside of ART clinic visits, and encourage providers to use best practices for counseling messages.

## INTRODUCTION

A growing body of research has identified a number of health systems and service delivery barriers to antiretroviral therapy (ART) adherence and retention in care among people living with HIV,^1^^,^^2^ particularly in high-prevalence low- and middle-income countries.^3^ Common challenges to delivering ART in resource-limited settings include overburdened clinical staff and facility congestion, resulting in constrained abilities to offer quality care.^4^^,^^5^ Although ART is provided free-of-charge in many low- and middle-income countries, the need for lifelong medication imposes burdens on clients, including the time and cost of frequent refill visits.^3^^,^^6^

Malawi has been heavily impacted by the HIV epidemic with a current prevalence of 9.4% and 740,000 adults currently on ART.^7^ Routine HIV care for clients who are stable is delivered using an integrated model in which all services (i.e., clinical evaluation and dispensing of ART and any other medications) occur in a single visit at a single location. Visits are not required outside of the routine ART visit unless the client has a change in clinical status that warrants closer follow-up.

In Malawi, clients on ART who are stable typically receive a 3-month supply of ART per visit, with additional exceptions to allow longer dispensing intervals for individuals with special circumstances (e.g., military service or traveling abroad). All individuals who have HIV in Malawi receive cotrimoxazole prophylaxis with ART, and a subset of people who live in high-burden tuberculosis districts also receive isoniazid preventive therapy.

Differentiated models of care (DMOC) may improve clients' adherence to ART and engagement in care by relieving their time- and cost-related barriers and may improve the capacity of health facilities and providers to care for clients who have been newly diagnosed or who require more support.^1^^,^^3^ Examples of ART DMOCs that have been implemented in sub-Saharan Africa include community-based ART groups, adherence clubs, fast-track refills, mobile clinics, and multimonth dispensing (MMD).^8^^–^^10^

The MMD model increases the quantity of ART received at one time and subsequently reduces the frequency of client facility visits.^11^ Guided by the U.S. President's Emergency Plan for AIDS Relief, longer dispensing intervals (up to 6 months of ART) are being implemented across Africa^1^^,^^7^^,^^12^^–^^14^; however, large-scale implementation experience with this model of care is lacking. Provider and client experiences with MMD have yet to be documented. As DMOCs, such as MMD, aim to affect all levels of the HIV service delivery cascade, understanding the experiences of both clients and providers is critical for examining the feasibility and acceptability of MMD and its potential for successful scale-up.

Varying Intervals of ART to Improve Outcomes for HIV (INTERVAL) is an ongoing cluster randomized controlled trial in Malawi and Zambia that is evaluating MMD for clients with HIV who are stable, a widely promoted DMOC.^14^ INTERVAL compares 3- and 6-month ART dispensing on the outcomes of retention, virologic suppression, and cost-effectiveness. The trial has been fully enrolled, and outcome data are currently being collected for the 1-year end point.

We performed a substudy to assess the experiences of providers and clients in Malawi comparing the 3- and 6-month dispensing arms. Our objective was to understand clients' and providers' perceived benefits and barriers of 3-month versus 6-month dispensing, with particular focus on areas of agreement and disagreement between providers' and clients' perceptions.

## METHOD

### Setting

The INTERVAL trial was conducted between May 10, 2017, and April 30, 2018, and included 15 health facilities in Malawi: 5 facilities randomized to 3-month dispensing, 5 facilities to 6-month, and 5 facilities to “standard of care” in which study interference was minimal and providers selected the amount of ART to dispense based on their clinical assessment and patient preferences. For this qualitative substudy, we included all 3- and 6-month health facilities from the INTERVAL trial (N=10). We did not include the standard-of-care facilities because they largely approximated 3-month sites. Health facilities were predominantly government and mission hospitals (80%) in the central and southern regions, and 60% of the study facilities had ART cohorts of more than 1,500 clients. INTERVAL health facilities were chosen based on their ability to enroll clients and to support MMD because of time and funding constraints; thus, they were generally larger than the average ART clinic in Malawi.

Clients in the INTERVAL trial received either 3 or 6 months of all medications to avoid the need for any additional refill visits during the year.

### Study Design

From June to August 2018, we conducted semi-structured in-depth interviews with a random subset of 62 enrolled INTERVAL clients from the 10 selected health facilities. Clients were eligible for in-depth interviews if they met INTERVAL trial eligibility as a stable ART client (see Box), were in either the 3- or 6- month study arm, and gave consent at baseline to be contacted for an interview at the end of the first year after enrollment. The 62 clients were stratified by study arm (32 from the 3-month arm, 30 from the 6-month arm) and gender to ensure equal representation. A total of 17 health care providers were randomly selected from the 10 chosen health facilities to be interviewed. Providers were eligible to participate in the study if they were clinical officers or nurses who directly prescribed ART at the selected study sites and had worked in the ART clinic at least once a week, on average, for a minimum of 6 months during the first year of study implementation. The study was approved by the Malawi National Health Sciences Research Committee and the Institutional Review Board at the University of California Los Angeles.

### Interview Guide Development

Interview guides were developed using McLeroy et al.'s socioecological model^15^ to elucidate relevant factors operating at the individual, interpersonal, community, and organizational levels. Clients were asked about the challenges and benefits of the amount of ART dispensed, including carrying, storing, sharing, and selling ART. Providers were asked how the dispensing interval at their site impacted workload and clinic efficiency. They were also asked about clients' satisfaction with the ART dispensing interval and whether clients reported any challenges with carrying, storing, and/or sharing ART. Both client and provider interview guides included probes to draw out information about additional non-ART facility visits (e.g., illness or family planning) during the year and the “ideal” ART dispensing interval. The interview guides were piloted with 10 clients and 2 providers before data collection began to ensure comprehensibility; they were refined based on feedback. Full interview guides are provided in the Supplement.

BOX **INTERVAL Trial Client Eligibility Criteria for Malawi**

Clients with HIV were eligible for the INTERVAL trial in Malawi if met the following inclusion criteria:

- 18 years of age or older
- On ART 6 months or longer
- On first-line ART regimen as defined by country-specific guidelines (efavirenz/lamivudine/tenofovir)
- No drug toxicity/tolerability issues within the prior 6 months
- Not without medication for >1 month during the last 6 months
- No active opportunistic infection suspected (including tuberculosis) and not treated for an opportunistic infection in the last 30 days
- Viral load <1000 copies/mL within the last 6 months
- If female, not pregnant or breastfeeding

Abbreviations: ART, antiretroviral therapy; INTERVAL, Varying Intervals of ART to Improve Outcomes for HIV

### Data Collection

In-depth interviews were independently conducted in private locations at the health facilities and ranged in duration from 20–60 minutes. Seven research staff members conducted interviews in the local language (Chichewa). All interviews were audio recorded after obtaining written consent from clients and providers. Clients were compensated for their transportation costs with a stipend of Malawi Kwacha 2,000 (approximately US$2.50).

### Data Analysis

Audio recordings were transcribed and translated to English. A preliminary codebook was developed for both interview types based on the socioecological model framework. Four investigators, 2 for client interviews (JH and CM) and 2 for provider interviews (KP and KM), piloted the codebook by independently reading and coding a randomly-selected subset of transcripts (8 clients and 6 providers). Through an iterative consultative process, each pair of investigators revised their respective codebook and repeated this process until there was high interrater reliability. All transcripts were coded in Atlas.ti version 8.3 using constant comparison, and coding disagreements were resolved by consensus. Analysis focused on agreement and disagreement between the 3- and 6-month arms and between clients and providers.

## RESULTS

The 62 clients interviewed (32 from the 3-month arm, 30 from the 6-month arm) were evenly divided by gender and had a median age of 41.5 years. The majority had disclosed their HIV status to their partner. Key differences in clients by study arm were in secondary educational attainment (31% in the 3-month arm versus 57% in the 6-month arm) and formal employment (25% in the 3-month arm versus 53% in the 6-month arm) (Table 1). The median number of ART pills dispensed to clients was 90 (interquartile range [IQR] 87–92) in the 3-month arm and 179 (IQR 175–182) in the 6-month arm, indicating a high level of adherence to the randomized dispensing strategy. One client in the 6-month arm met the national clinical criterion for treatment default (out of care for >60 days) during the study follow-up period of 1 year.

**TABLE 1.** Demographic Characteristics of Clients With HIV on ART Participating in In-Depth Interviews in 10 Health Facilities in Malawi (N=62)

	INTERVAL Trial Arm	
	Clients Receiving 3 Months of ART (n=32)	Clients Receiving 6 Months of ART (n=30)
**Gender**, No. (%)		
Female	16 (50)	16 (53)
Male	16 (50)	14 (47)
**Age**, years, median (IQR)	40 (35–47)	43 (37–50)
**Marital status**, No. (%)		
Married	25 (78)	26 (87)
Unmarried	7 (22)	4 (13)
**Disclosure of HIV status to primary sexual partner**, No. (%)		
Yes	26 (81)	26 (87)
No	1 (3)	0 (0)
No primary sexual partner	5 (16)	4 (13.3)
**Household size**, median (IQR)	5 (4–6)	5 (3–7)
**Number of children**, median (IQR)	2 (1–3)	1 (1–3)
**Employment**, No. (%)		
Formal employment	8 (25)	16 (53)
Informal employment	18 (56)	12 (40)
Not working	6 (19)	2 (7)
**Education**, No. (%)		
No education	3 (9)	2 (7)
Primary	19 (59)	11 (37)
Secondary or higher	10 (31)	17 (57)

Abbreviations: ART, antiretroviral therapy; INTERVAL, Varying Intervals of ART to Improve Outcomes for HIV; IQR, interquartile range.

We interviewed 17 ART providers (8 from the 3-month arm and 9 from the 6-month arm); of these, 9 were women. The providers had a median age of 35 years and nearly all had been dispensing ART for 4 or more years. The majority of providers were nurses (n=14), and the remaining providers were clinical officers (n=3).

### Sharing and Selling of ART

When asked about sharing or selling ART, the vast majority of clients in both arms reported that they did not share ART (97%) and cited the importance of maintaining their own drug supply.

*How will I share? If the person wants drugs, he/she should go to the hospital to do that. We don't share (ART) with each other like panado [acetaminophen]—this is not panado.* —Female, 44 years old, 6-month arm

The majority of clients reported that they did not share or sell ART.

Clients cited several reasons they did not share or sell ART: (1) they think that the drugs are precious because they are lifesaving, (2) they get different ART medications, (3) they have been instructed by health workers not to share, and (4) they will run out of pills sooner and may have problems at refill appointments.

*If I sell a bottle it means I have wasted 1 month. So, when I go to the hospital, what lie will I tell the doctor? … I can't sell a bottle of drugs because it will mean that I'm selling my life.* —Female, 37 years old, 6-month arm

Several clients said that when someone had asked them for medicines, they did not share or sell pills, but they helped in other ways such as escorting the individual to the health facility or gifting them with other items like money or flour.

*She came to me and said, “I'm too shy to go to the hospital. Share your drugs. If people see me receiving the drugs, they will laugh at me.” I told her, “You are saying that they will laugh at you, but everyone keeps his/her own life. Let's go to the hospital. I will escort you. If you think it's far, I will also use my transport money.”* —Female, 30 years old, 6-month arm

In contrast, providers in both the 3- and 6-month arms raised concerns about pill sharing, primarily among couples. Commonly cited scenarios included sharing 1 bottle between 2 partners, sharing when a partner runs out of their medication, and sharing when a partner is unable to return to the ART clinic for a refill due to work or family reasons.

*[It's] very common [sharing of medication]. You find someone has missed their appointment date. When you ask them, they tell you, “Yes, but there is never a day that I have missed without taking drugs.” When you ask how, you will hear them say, “I was taking my wife's drugs.”* —Clinical Officer, 3-month arm

Less common among the providers were concerns of sharing ART with friends and in the broader community, although a few examples were provided in both arms.

*A client said, “I forgot mine [ARVs].” And the other said, “You shouldn't forget,” as she took them [ARVs] from her wrapper. Seeing this, you know that these people are sharing drugs.* —Nurse, 3-month arm

Clients and providers were unable to provide examples of selling ART. Both clients and providers questioned the benefit of selling when asked because ART is freely available. Clients in the 6-month arm did not report added pressure or desire to share or sell ART despite having an increased supply of medication.

*I have never heard [of selling], but I can say it would be difficult for a person to buy something that can be acquired easily [for free]*. —Clinical Officer, 3-month arm

Both providers and clients had heard rumors of alternative uses of ART, such as mixing it with beer and spirits to improve taste and potency and/or feeding ART to livestock to help growth, but no one reported any personal or direct experience with these uses of ART.

*What people say is that they want the beer to be sour and that people should get drunk fast [if ART is added]. I don't have the proof that people do that.* —Male, 46 years old, 6-month arm

### Storage of ART

Approximately half of the providers in both arms expressed concerns about clients' ability to store an increased supply of ART. Although providers gave limited specific examples, one provider remembered a client who had reported his ART was destroyed because it was stored close to where he built his fire in his home.

*Today, a patient showed me drugs that have completely melted. That person was given 3 months' supply. When I asked, he said, “Where I store my drugs, I also make fire.” So, when I give him 6 months' supply, if the 3 months' supply has melted, what will happen to the other?* —Clinical Officer, 3-month arm

Half of the providers in both 3- and 6-month arms expressed concerns about clients' ability to store a larger supply of ART, but clients reported no challenges.

When we asked clients about storage, none reported challenges with storing the 3- or 6-month supply. In both arms, clients frequently mentioned that protecting ART from damage (e.g., water or sun exposure) and keeping medication out of children's reach were considerations with medication storage. Additionally, clients reported that disclosing their HIV status to their household members facilitated storage of ART because they did not need to hide their supply at home and they could depend on others to assist in keeping them safe.

*My husband also receives the drugs, so I don't hide [them]. My husband is also aware of the amount of drugs I have.* —Female, 54 years old, 6-month arm

*Drugs are dangerous to children. Children can't recognize them. They can get them [the drugs] and eat [them], and this can cause an accident. So, it's like hiding them [the drugs] and taking care of them [the children] at the same time.* —Female, 36 years old, 6-month arm

All clients were asked if they had lost or misplaced their ART or if it had been stolen during the study. Only 1 client in the 3-month arm said they had lost their ART supply. When asked about keeping ART safe, clients strongly voiced that they protected their ART because it was lifesaving.

*No, I have never lost any. I take care of them because it's my whole life.* —Female, 53 years old, 3-month arm

### HIV Status Disclosure and Stigma

The majority of clients on ART in both arms reported having disclosed their HIV status to their primary sexual partner (96% in the 3-month arm and 100% in the 6-month arm). Providers did not discuss stigma and disclosure challenges in general or related to 3- versus 6-month dispensing. No clients in either arm reported unwanted partner disclosure related to ART supply. However, for some clients, carrying ART from the health facility was associated with a fear of unwanted disclosure to community members. Clients in both 3- and 6-month arms reported instances of being mocked for carrying ART bottles, particularly when they carried their medicines in plastic bags.

*One day, they [community members] saw me carrying the bottles. They said a lot of bad things like, “Look at him, he is coming from the hospital carrying [ART], he is a fool, he is about to die.”* —Male, 44 years old, 3-month arm

Clients in the 6-month arm reported that although carrying the increased drug supply was not a burden, they changed their routine to accommodate it by adopting sturdier and more private bags like backpacks and cloth bags. Providers also noted that clients in the 6-month arm had changed their carrying methods.

*When they changed me to 6 bottles, it was difficult to put 3 bottles in 1 pocket and the other 3 bottles in another pocket. That is why I thought of taking a bag to carry the medicine.* —Male, 47 years old, 6-month arm

### Benefits of Multimonth Dispensing

Clients in the 6-month arm reported several benefits of having less frequent visits to the health facility: (1) decreased direct costs in transportation; (2) decreased indirect costs in lost wages; (3) less time spent traveling to the health facility, particularly if they lived far away; and (4) less time waiting.

Clients reported that the 6-month dispensing interval was very helpful in alleviating these constraints.

*I'm able to work for our daily needs and have time to rest and find food for the day. I see it as a good thing compared with back then [when receiving standard of care] when you had to cancel plans so that you can go to the hospital to get drugs.* —Female, 38 years old, 6-month arm

Clients in both arms mentioned feeling a greater sense of freedom and normalcy with fewer ART facility visits; this benefit was particularly evident among those in the 6-month arm. Examples of this freedom included being able to avoid unwanted HIV status disclosure due to fewer facility visits and having the freedom to prioritize personal and work endeavors.

*I forget that I am a patient. I don't often have concerns that today or tomorrow I should go and get drugs. When I notice how much time I have, I do my work without any problems. When the appointment date is due, I come here.* —Male, 39 years old, 6-month arm

*I can tell the difference between the time that I was getting 3 bottles because now I come here fewer times. Since I am working, it is not good to be excusing yourself, and sometimes they [employers] don't respond positively, saying, “You are fond of excuses.” I think it is very helpful to be getting 6 bottles.* —Female, 39 years old, 6-month arm

Clients in both study arms mentioned feeling a greater sense of freedom and normalcy with fewer ART facility visits.

Providers in the 6-month arm reported that longer ART dispensing periods also helped the health facility by alleviating clinic congestion resulting from large numbers of stable clients needing refills. Providers in the 3-month arm spoke about how 6-month dispensing would be beneficial for their workload.

*But to me the option that can reduce my workload is the option of 6-months dispensing. And tell the patient that please if they have any problems, they can come and meet me.* — Nurse, 3-month arm

### Additional Facility Visits

Seventy-two percent of clients reported visiting a health facility during the study period for intercurrent illnesses including cough, headache, and skin rashes (77% in the 3-month arm and 66% in the 6-month arm). Only 2 of the 31 female respondents (both in the 3-month arm) reported returning to the ART facility for family planning during the year.

Compared to the 3-month arm, a lower percentage of clients in the 6-month arm reported visiting a health facility for intercurrent illnesses.

When asked about return visits, a subset of providers associated the 6-month dispensing strategy with poor client health-seeking behavior between appointments.

*Those who are having a problem are forced to stay home until the [refill] date comes; that's what I have observed. Maybe they have developed a cough, and they are a TB [tuberculosis] suspect, but they don't come until they reach their appointment date.* —Nurse, 6-month arm

### Ideal ART Supply

Both clients and providers were asked about the ideal ART dispensing interval. Although providers raised concerns about clients' delayed health-seeking behaviors, nearly all providers chose a 6-month interval. They cited reasons including reduced congestion of clinics, reduced provider workload, and improved ability to care for clients who are newly initiated and unstable/ill. They also reiterated the benefits for clients such as reduced burden of accessing the clinic frequently.

Clients' ideal ART dispense interval ranged from 4 to 12 months, with approximately half choosing either 6 months (n=17) or 12 months (n=16). The reasons provided related to the benefits of decreased clinic visits that bring increased freedom and financial savings.

*Six bottles would be much better. [Six-month dispensing] would help doctors have enough time to rest. Not only that, it would ease our mobility challenges and give us enough time to rest.* —Female, 35 years old, 3-month arm

*I would be happy if they gave 6 months', even a year [supply]. It would do me a lot of good. When your appointment date is due, you plan and raise transport money. But maybe the day comes, and you don't have transport [money]. I farm during rainy season, and it's hard to stop and go out and look for transport money. You can even go into debt just so you can go to the hospital and get drugs.* —Female, 38 years old, 6-month arm

A summary of areas of client and provider similarities and differences is provided in Table 2.

**TABLE 2.** Comparison of Providers' and Clients' Perceived Challenges and Benefits of 6-Month ART Dispensing Interval, 10 Health Facilities in Malawi

Socioecological Model Theme	Client	Provider
**Individual**		
Sharing ART	Only 1 client reported sharing; all others did not share	Considered a common problem, particularly sharing among partners
Storage of ART	No reported challenges	Perception that clients have challenges storing increased ART supply
**Communal**		
Unwanted HIV status disclosure to community	Reported minimal challenges with easy adaptation strategies to avoid unwanted disclosure	Did not report challenges with pill carrying but perceived adaptive behaviors (i.e., using different bags)
Selling ART	No reports of personal experience with selling	No concern about selling
**Organizational**		
Visits for ART refills	Reduced number of visits Reduced cost Increased time and ability to attend to family and work demands	Reduced clients' costs and time Reduced providers' workload and clinic congestion Improved ability to see newly initiated/unwell clients
Visits for acute health needs	Reported returning for acute illnesses frequently	Perceived delays in clients' health-seeking behaviors

## DISCUSSION

In this qualitative substudy of the INTERVAL trial, long dispensing intervals were highly acceptable to clients and providers, with an ideal dispensing duration of 6 to 12 months. These findings are both valuable and timely in addressing potential implementation concerns of the acceptability of 6-month dispensing as a DMOC. Decreased clinic visits emerged as the strongest benefit of extended refill intervals for both clients and providers. This finding echoes that of other studies that have found that minimizing refill visits is highly attractive to both clients and providers.^16^ For clients, the 6-month dispensing interval provided time and financial savings related to a less frequent visit schedule. This finding aligns with many other studies that identify health-seeking costs—long wait times, expensive transport, and lost wages—as major barriers to successful ART adherence.^3^^,^^17^^–^^19^ Additionally, several clients in the 6-month arm noted that reduced clinic visits improved their ability to keep their HIV status private to the community and contributed to feelings of normalcy because they were spending less time seeking health services.

Longer dispensing intervals were highly acceptable to clients and providers.

Benefits of decreased clinic visits also extended to the health system level. Providers reported decreased workload and improved ability to see clients who are unstable. Findings from other DMOCs, including fast-track refills and community-based ART groups, also show improved operational efficiency at the clinic level by reducing patient overcrowding and staff workload.^5^^,^^10^^–^^12^

Providers reported that longer dispensing intervals decreased workload and improved their ability to see clients who are unstable.

None of the clients in the 3-month arm reported benefits associated with this interval that suggested it was superior to 6-month dispensing. There were no obvious trends in our analysis to suggest that women had outlying barriers related to 3- and/or 6-month dispensing when compared to men.

Although clients and providers agreed about the acceptability of MMD, they disagreed about certain aspects. Providers perceived challenges and voiced concerns around clients' ability to handle the larger ART supply. Providers reported that sharing ART was common, but clients denied sharing and provided examples of alternative methods of support (i.e., transport, accompaniment) for those needing ART. Studies evaluating MMD have not reported instances of clients sharing ART due to the extended supply. However, sharing has been documented with other prescription medications, including antibiotics and antihypertensives.^20^ It is possible that clients underreported sharing due to social desirability bias because ART providers strongly discourage it.

Medication storage was another provider concern that was not borne out in the client interviews. High rates of HIV disclosure (98%) facilitated ART storage among clients in our study. Findings may be different in a population with lower rates of disclosure. If 6-month dispensing is taken to scale, providers should review proper medication storage guidelines with clients and should suggest ways to ease burdens related to carrying and storing an increased drug supply.

Clients in both the 3- and 6-month arms mentioned that the value of ART was a reason they did not sell or share ART. The 6-month interval did not incentivize selling or sharing. Although the literature indicates that financial stress can influence ART adherence due to lack of money for transport to clinic or inability to step away from income-generating activities for refill visits,^2^^,^^18^^,^^21^ there is scarce published evidence of selling ART for economic improvement. As clients and providers mentioned, there is a limited market for selling ART because medications are provided for free in most settings. It is possible that individuals underreported selling, but research staff were trained to emphasize confidentiality of responses and to build rapport to facilitate an honest discussion.

Finally, although most (72%) clients reported utilizing health services for intercurrent illnesses between their longer-duration ART refill visits, providers perceived 6-month dispensing to be associated with delayed presentation of acute illnesses. A study evaluating the impact of spaced clinic appointments for clients with HIV (6 months versus 1 month) found that 30% were removed from 6-month dispensing due to having unstable medical conditions such as decreased CD4 counts, ART regimen changes, pregnancy, nonadherence, and drug toxicity.^22^ If 6-month dispensing is taken to scale, clients should be counseled about coming to the ART clinic without delay for any new health issues. Providers should receive appropriate training about when to shift clients to shorter intervals due to poor adherence/virologic failure, side effects, after regimen changes, or during pregnancy.

Although we did not ask clients specifically about family planning visits at other types of facilities nor about community- or pharmacy-based family planning services, we found low reported utilization of family planning services at the ART clinic aside from refill visits. Thus, family planning service use may have been underreported (all women of reproductive age in the 6-month arm were counseled on the importance of regular family planning visits upon study enrollment). Future program implementation and research should focus on improved access to family planning under 6-month dispensing and/or other DMOCs that reduce clients' interactions with health care providers.

### Limitations

Our study has several limitations. Clients were recruited for interviews if they could be reached by phone; this potentially resulted in selection bias based on socioeconomic status. Because the INTERVAL study is an unblinded clinical trial, both clients' and providers' responses may be subject to social desirability bias. We did not specifically ask providers how they adjusted their counseling with clients in the 6-month dispense interval arm. All but 1 of the 62 randomly selected clients were retained in care based on their randomly assigned ART dispense interval. Clients who did not stay on their assigned dispensing interval, defaulted from care, or were taken off of their interval for clinical reasons were not represented. Similarly, providers interviewed for the study may not necessarily reflect the views of all health care workers at the study sites.

## CONCLUSION

Both clients and providers perceived 6-month ART dispensing as highly feasible and acceptable, citing benefits such as reduced cost of transport for clients, increased time for income-generating activities for clients, and improved clinic efficiency for providers. Further research is needed on encouraging client health-seeking behaviors for acute illnesses and use of family planning services for clients on 6-month dispensing intervals. If 6-month dispensing is scaled in high-burden HIV settings, ongoing evaluation should be performed to include both client and provider perspectives on the benefits and challenges of this DMOC.

## Supplementary Material

19-00286-Hubbard-Supplement.docx

## Notes

**Peer Reviewed**

**Funding:** Funding was provided by the U.S. Agency for International Development and the U.S. President's Emergency Plan for AIDS Relief under Cooperative Agreement [AID-OAA-A-15-00070].
