# Supplementary material for: A Qualitative Assessment of Provider and Client Experiences With 3- and 6-Month Dispensing Intervals of Antiretroviral Therapy in Malawi
Source: Glob Health Sci Pract. 2020 Mar 30;8(1):18–27. doi: 10.9745/GHSP-D-19-00286 (PMC7108939; doi:10.9745/GHSP-D-19-00286)
Supplement: 19-00286-Hubbard-Supplement.docx [file 19-00286-Hubbard-Supplement.docx]

**19-00286-Hubbard-Supplement**

**In-Depth Interview Guides**

**CLIENT POST-INTERVENTION INTERVIEW**

INTERVAL STUDY MALAWI

1. Please describe how you feel about your current amount of ART supply [3 or 6 months].

2. Have you faced any concerns or challenges while transporting your ART or other HIV medications from the clinic to your home (*probe: any difficulty carrying the amount of medications; concern around unwanted disclosure because of being seen carrying medications from clinic to home*)?

- 1. If so, please describe these. Were you able to overcome these difficulties, and if yes, how?

3. Where do you currently store your bottles of ART? Have you faced any challenges with storing your ART (*probe: difficulty finding space at home, difficulty keeping your HIV status private because of storing/keeping the medicines at home?*)

a. If so, please describe these. Have you been able to overcome these challenges? If yes,

how?

4. Have any of your ART bottles been lost for any reason, stolen, or missing?

a. If so, please explain what happened.

b. Tell me about your experience when you went to the facility to get more/replacement medication.

5. In the last year have you ever felt pressure to share your ART with other people (family member, friend, neighbor)? If yes, please describe the situation (with whom and why).

a. If you did share: Once you gave ART to someone else, what happened (*prompt – did you start skipping days? Did you run out of medication*?)

b. What happened when you went back to clinic?

6. Without mentioning names, have you ever heard of people with HIV who sell their ART for money?

a. If so, what are the reasons for selling their drugs? Can you describe more about how this works (*prompt:* *where do they sell? who typically buys ART?*).

7. In the last year have you felt any pressure to sell your ART? If yes, please describe the situation. If you sold your medications, can you talk more about the situation (to whom, how did you use the money?)

a. If you did sell your medications, what happened (*prompt: did you run out, did you start skipping days*?)

b. What happened when you went back to clinic (*prompt: did you have challenges?)*

8. In the last year, apart from your ART refill appointments, how many times did you go back to the ART clinic for problems related to your own health? Can you tell me more about the problems that caused you to go back to clinic apart from ART refills (fever/malaria, headache, family planning, other)?

9. What would be the ideal amount of ART you would like to get each time you come to the facility (*Probe- interviewer can offer anywhere from 1 to 12 months*)?

a. Why did you pick this amount -- what are the extra benefits and/or challenges?

10. If the amount of ART (number of bottles) you receive changed from last year to this year, can you describe any extra benefits or extra challenges associated with the amount you get now compared to the amount you used to get?

**PROVIDER POST-INTERVENTION INTERVIEW**

INTERVAL STUDY MALAWI

1. For patients who have been getting [3 or 6] months of ART in the INTERVAL study during the last year, can you describe:
   1. The **experience** patients have had receiving that amount of ART, both good and bad.
      1. Probe: your **observations** related to the patients receiving the amount of ART?
      2. Probe: any **perceptions/thoughts** you have related to receiving the amount of ART?

(*Overall probes:* *challenges with carrying ART and other meds, challenges storing their ART at their home, better adherence to ART, better attendance at clinic appointments, etc.*)?

- For any challenges or problems that patients experienced, can you describe specific ways in which challenges were overcome?

1. Do you think the dispensing interval of [3 or 6 months] has any benefits or challenges for your work or experiences **as an ART provider**? Please describe (*Probe: less or more busy seeing patients; fewer or more patient complaints or praise; fewer or more concerns about patient adherence/retention*)?
2. Do you think the dispensing interval of [3 or 6 months] has any other benefits or challenges **for the ART clinic or facility**? Please describe. (*Probe: any impact on the clinic resources, personnel, clinic less busy/congested, more busy/congested, drug expiration or supply problems*)?
3. In the last year, have you noticed any problems with the ART supply chain such as stock outs of 5A or CPT or INH? If so, please describe:
   1. What happened?
   2. Why?
   3. What was the impact on your ability to provide medications to patients?
   4. Any negative impact on patients?
   5. How were the problems overcome?).
4. In the last year have you noticed that any ART has been near the expiration date at the time it was dispensed, such that they would expire before the patient had a chance to use the entire supply? If yes, can you talk about what happened and how you handled this situation? (*Probe:* *worked with pharmacy to get a supply that wouldn’t expire, gave the patient a smaller drug supply, other?*)
5. We have heard that patients sometimes sell or barter their drugs. Without mentioning names, do you know if patients sell or use their ART for personal gain (probes: exchange or sell for chickens, or cows, or giving a bottle of ART in exchange for a liter of home brew beer)?
   1. Specifically for INTERVAL patients, have you heard this being the case?
      1. If yes, can you describe more about the circumstances?
      2. Note - at 6-month sites ensure providers comment on whether selling changed with the introduction of 6-month supply.

(*Probe:* *how common is this? why does this occur? Who buys ART? Do you give refills if patients come back early needing refills?*).

1. We have also heard that patients sometimes share their ART with family and friends. Without mentioning names, do you know how commonly patients share their ART?
   1. Specifically, for INTERVAL patients, have you heard this being the case?
      1. If yes, can you describe more about the circumstances?
      2. Note - at 6-month sites ensure providers comment on whether selling changed with the introduction of 6-month supply.

(*Probe:* *how common is this? why does this occur? Who buys ART? Do you give refills if patients come back early needing refills?*).

1. In instances where an INTERVAL patient runs out of their ART early [3 or 6 months] and come to the clinic early for a refill, how to you handle the situation? What is your response? Do you know why they run out early (if not previously mentioned above)?
   - 1. Note - at 6-month sites ensure providers comment on whether this changed with the introduction of 6-month supply (whether people seem to run out more commonly or less commonly)
2. Do you know of health care providers who ever sell ART? If yes, can you describe more about the circumstances? (*Probe:* *how common is this? why does this occur? Who buys ART? What are the consequences for the clinic, if any?*)
   - 1. Do you think providers will be more likely to sell ART with 6-month dispensing?
     2. Do you think providers feel pressure to receive favors from patients in order to allow them to get 6 months of ART? What sorts of favors?

10. What do you think is the ideal amount of ART that stable HIV patients should be given (*interviewer should offer 1­–12 months*)? Why do you think this is the ideal amount?

NOTE: As a reminder, for the purposes of this conversation, stable patients are adults (age 18 and older) who are on first line ART, do not have challenges with adherence, do not have other active medical problems that require close follow-up (infections such as TB), and are not pregnant or breastfeeding.

1. If you had the opportunity to pick ONE option from a list of options of how people receive their ART, what do you think is the best option:
   1. Fast track refills – patient sees the clinician, gets 3-month refill, returns at 3 months but is ‘fast-tracked’ (skips the queue and does not see the clinician) for a refill and returns to see the clinician 3 months later
   2. Community ART groups or “CAGs” – single members of a larger group come to pick up drugs for the whole group, each patient only seeing the provider 1–2 times a year depending on the size of the group
   3. 6-month dispensing (facility-based)
   4. 3-month dispensing (facility-based)

Why would they choose that particular option? Are there other options we have not discussed that you would recommend?
